# Evidence for enhancer activity in intron 1 of *TNFRSF1A* using CRISPR/Cas9 in human induced pluripotent stem cell-derived macrophages

**DOI:** 10.1038/s41598-025-18077-9

**Published:** 2025-10-07

**Authors:** Julie A. Osgood, Andrew C. Brown, Katie L. Burnham, Olga Mielczarek, Gabriele Migliorini, Chwen Tay, Ping Zhang, Madeleine H. Palmer, Ben Davies, Sally A. Cowley, Julian C. Knight

**Affiliations:** 1 Centre for Human Genetics, University of Oxford, Oxford, UK; 2 NIHR Oxford Biomedical Research Centre, Oxford, UK; 3 Nuffield Department of Medicine, Chinese Academy of Medical Sciences Oxford Institute, University of Oxford, Oxford, UK; 4 The Francis Crick Institute, London, UK; 5 Sir William Dunn School of Pathology, James and Lillian Martin Centre for Stem Cell Research, University of Oxford, Oxford, UK

**Keywords:** TNFRSF1A, Epigenomics, Functional genomics, Transcriptomics, Gene regulation, CRISPR, Gene regulation, Genetics research

## Abstract

TNFα is a common drug target in the treatment of autoimmune diseases, with pro-inflammatory functions that are primarily mediated through its receptor, TNFRSF1A. *TNFRSF1A* has been genetically associated with many immune-mediated diseases including ankylosing spondylitis, multiple sclerosis, and inflammatory bowel disease. Many of the genetic variants within or near *TNFRSF1A* that have been associated with disease through genome-wide association studies (GWAS) lie in non-coding regions of the genome. Understanding the functional consequences of these genetic variants is limited by incomplete understanding of *TNFRSF1A* gene regulation, including for specific cellular contexts relevant to inflammation and immunity such as macrophages. This work used CRISPR/Cas9 in human induced pluripotent stem cells followed by differentiation into macrophages to investigate putative regulatory elements in the *TNFRSF1A* gene locus. Through gene editing, with functional genomic readouts including the assay for transposase-accessible chromatin using sequencing (ATAC-Seq), chromatin immunoprecipitation with sequencing (ChIP-Seq), and RNA-Seq to assess the consequences of these edits, we present evidence for an enhancer of *TNFRSF1A* contained within an intron of the gene. Understanding gene regulation and the genomic context in which GWAS variants lie could bring us closer to deconvoluting the genetic basis of common disease aetiology and uncover effective drug targets.

**Supplementary Information:**

The online version contains supplementary material available at 10.1038/s41598-025-18077-9.

## Introduction

The tumor Necrosis Factor Receptor Superfamily Member 1A (*TNFRSF1A*) gene encodes a 55KDa transmembrane receptor for tumor necrosis factor alpha (TNFα) that is constitutively expressed on almost all cell types^1^ and mediates most of the pro-inflammatory functions of TNFα^2–5^. Binding of TNFα to membrane-bound TNFRSF1A initiates a signaling cascade that results in apoptosis of the cell, or in the activation of NF-κB to increase the expression of pro-inflammatory genes. Conversely, soluble TNFRSF1A can bind to free TNFα and act as a natural inhibitor by preventing TNFα binding with membrane-bound receptors^6^. Consistent with the known role of TNFRSF1A as an important regulator of TNFα and the inflammatory response, the gene locus has been associated with multiple immune-mediated disorders. Genome-wide association studies (GWAS) have uncovered associations between *TNFRSF1A* and ankylosing spondylitis^7^, primary sclerosing cholangitis^8^, ulcerative colitis^8^, primary biliary cholangitis^9^, psoriasis^8^, Crohn’s disease^10^, noise induced tinnitus^11^, and multiple sclerosis^12–14^. *TNFRSF1A* has also been associated with cancer, and mutations in the gene^15,16^ are the cause of Tumor necrosis factor Receptor Associated Periodic Syndrome (TRAPS).

These genetic associations with autoimmune and inflammatory diseases further support a role for *TNFRSF1A* in immune-mediated disorders, consistent with the demonstrated efficacy of TNF inhibitors as a common treatment option for a variety of these diseases^17–21^. However, despite many associations with immune-mediated disorders, *TNFRSF1A* gene regulation is not well understood. It is thought that its promoter may be constitutively active due to a CCAAT/enhancer binding protein (C/EBP) binding motif^22,23^. The promoter also contains binding sites for AP-1 transcription factor, which modulates gene expression in response to viral and bacterial infections, and NF-κB which upregulates *TNFRSF1A* gene expression during an inflammatory response^23,24^. *TNFRSF1A* can also be regulated through epigenetic modifications^25,26^. Recent studies have reported that TNFα also plays a role in *TNFRSF1A* gene regulation and that TNFα exposure in cell lines can alter the abundance of specific *TNFRSF1A* isoforms and dictate cell numbers expressing the receptor^27,28^.

Macrophages are key players in the innate immune system and are involved in chemotaxis, phagocytosis, and antigen presentation^29^. Macrophages are prominent producers of TNFα and are also responsive to it, making them a relevant cell type to study *TNFRSF1A* gene regulation^30^. The aim of this work was to use CRISPR/Cas9 (clustered regularly interspaced short palindromic repeats/CRISPR associated protein 9) genome engineering for a functional interrogation of a candidate enhancer region in human induced pluripotent stem cells (iPSCs), followed by differentiation into macrophages, with functional genomic readouts including the assay for transposase-accessible chromatin using sequencing (ATAC-Seq), chromatin immunoprecipitation with sequencing (ChIP-Seq), and RNA-Seq used to assess the consequences of these edits.

## Results

### Epigenomic landscape of *TNFRSF1A* indicates a putative regulatory element in intron 1

**Fig. 1:**
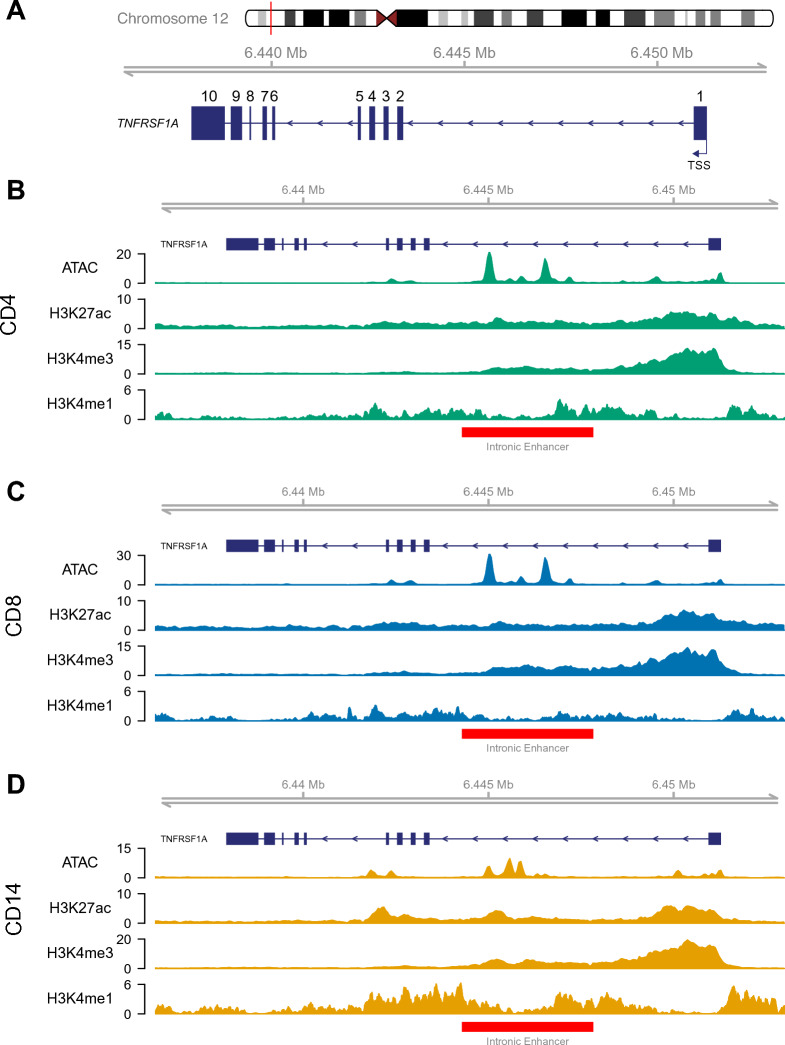
Epigenetic landscape of the *TNFRSF1A* locus. (**A**) Schematic showing the *TNFRSF1A* gene with the Intronic Enhancer highlighted in red. The numbers denote exons and the transcription start site (TSS) is indicated. Genomic view of the results of ATAC-Seq, H3K27ac ChIP, and H3K4me3 ChIP in (**B**) CD4 + primary cells (**C**) CD8 + primary cells and (**D**) CD14 + primary cells. Data for the primary cells were taken from healthy controls as described in Brown et al.^31^.

We first performed an in silico analysis of the *TNFRSF1A* gene locus to identify putative regulatory elements for investigation in three disease relevant cell types, CD14 + monocytes, CD4 + and CD8 + T cells. We interrogated ChIP-Seq and ATAC-Seq data for purified populations of these cells isolated from peripheral blood of healthy donors we have previously generated^31^ and found that the *TNFRSF1A* promoter is accessible and active in all three primary cell types (as indicated by the ATAC-Seq peaks and histone marks, H3K27ac, H3K4me3 and H3K4me1) (Fig. 1). We identified ATAC-Seq and H3K27ac and H3K4me1 ChIP-Seq peaks that would be consistent with the presence of an intronic enhancer in all three cell types. These findings are also seen with ENCODE data^32^ for this region showing open chromatin (based on DNase I hypersensitive sites and ATAC-Seq) in CD4 + , CD14 + , CD20 + , and K562 cells together with histone marks (H3K27ac, H3K4me1) indicative of an enhancer in GM12878 and K562 cells (Fig. S1), and annotation as a predicted enhancer by ORegAnno^33^. Furthermore, the region shows evidence of transcription factor binding, including *MYC*, *IKZF1*, and *GATA2*, based on ChIP data from ENCODE^32^, and conservation across 44 vertebrate species, as annotated by the phastCons program^34^. These findings indicate a putative enhancer within the first intron of *TNFRSF1A*, which here we refer to as ‘Intronic Enhancer’ (Fig. 1). There were subtle differences between the cell types in the open chromatin arrangement, suggesting different functions in CD4/CD8 + and CD14 + cells. In CD14 + cells there are three large open chromatin peaks across the region, all potentially functioning together to work as an enhancer or multiple enhancers aligned sequentially. To further understand the role of this intronic region, a luciferase assay was utilised to assess its ability to act as an enhancer.. This showed approximately a twenty-five fold increase in activity over a minimal promoter (Fig. S2).

### iPSC-derived macrophage cell model generation

**Fig. 2:**
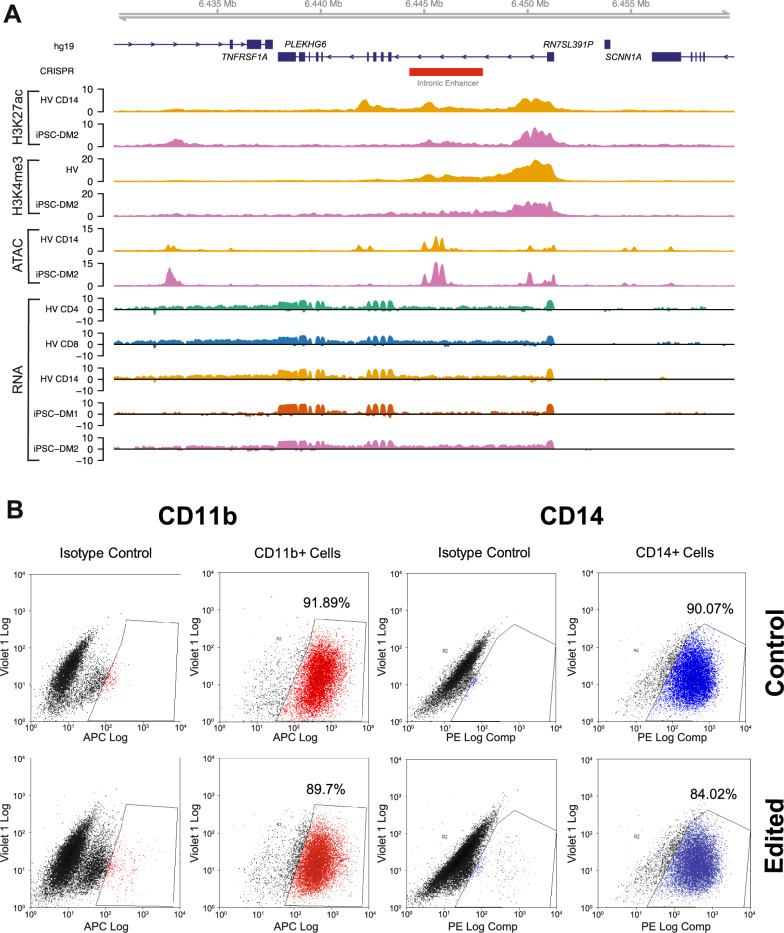
RNA and cell surface marker expression of iPSC-derived macrophages (**A**) Functional genomics readouts for primary immune cells and iPSC-derived macrophages. ChIP-Seq tracks for H3K27ac and H3K4me3 histone marks in CD14 + cells and iPSC-derived macrophages (iPSC-DM), ATAC-Seq tracks for CD14 + cells and iPSC-DM, RNA-Seq tracks for CD4 + , CD8 + , and CD14 + primary cells and iPSC-DM. iPSC-DM1 refers to the iPSC-derived macrophages produced and experimented on in this work. iPSC-DM2 refers to another set of iPSC-derived macrophages, produced independently, but from the same iPSC line, SFC840-03-03. (**B**) FACS profile of iPSC-derived macrophages. A single cell line each of iPSC-derived macrophages (CRISPR Control and Edited) following FACS analysis with CD11b (red) and CD14 (blue) antibodies. Over 90% of the cells were CD11b + and CD14 + for controls and over 84% for edited, indicating that the iPSCs were successfully differentiated into macrophages. The other cell lines examined shared a similar profile.

We next validated iPSC-derived macrophages^35^ as a relevant model for in vivo* TNFRSF1A* gene regulation by comparing functional genomics readouts between CD14 + primary cells and iPSC-derived macrophages. RNA-Seq, ATAC-Seq, and ChIP-Seq were carried out on iPSC differentiated macrophage cell lines and showed that the epigenomic landscape of the *TNFRSF1A* locus in iPSC differentiated macrophage cell lines was similar to primary CD14 + monocytes (Fig. 2A). To validate the macrophage phenotype, flow cytometry was performed. CD11b and CD14 were used as markers of successful differentiation into macrophages as they are both cell surface markers, strongly expressed by macrophages and are not expressed by iPSCs. After differentiation, CD11b and CD14 were expressed, indicating that the iPSCs were successfully differentiated into macrophages in both the control and edited cells (Fig. 2B).

### Deletion of putative enhancer region using CRISPR/Cas9

**Fig. 3:**
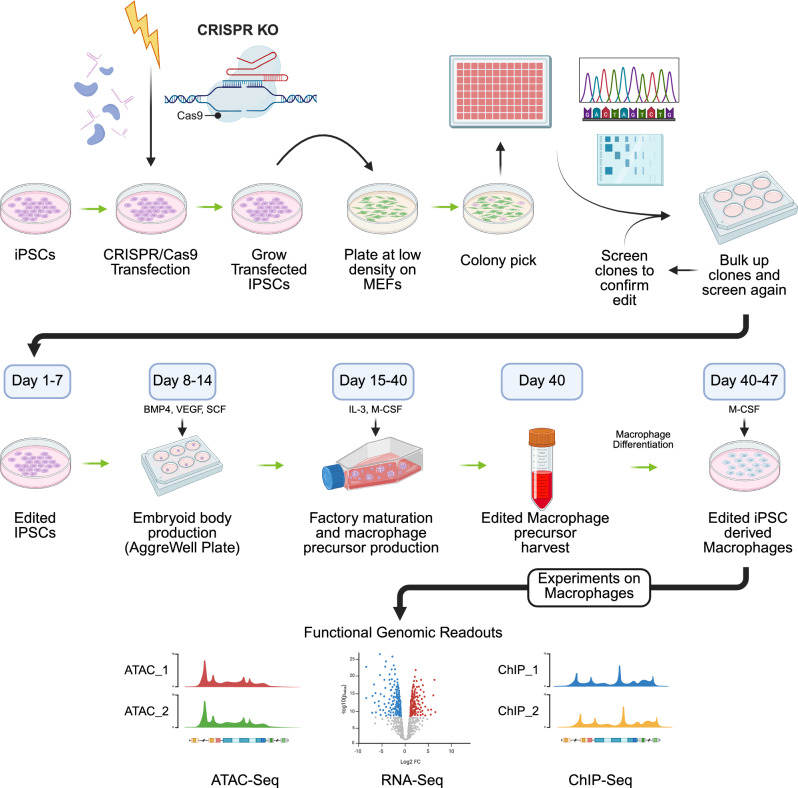
CRISPR/Cas9 and macrophage differentiation process. Overview of the CRISPR/Cas9 and macrophage differentiation process beginning with IPSCs, followed by genome editing and screening, differentiation into macrophages, and finally ending with functional genomic readouts. Created with BioRender.com.

CRISPR/Cas9 genome editing was carried out to delete the genomic region spanning the putative enhancer, using two guide RNAs (gRNAs). The putative Intronic Enhancer deletion region is 3346 bp (chr12:6444399–6447744, hg19) and three iPSC clones were generated with the deletion. The zygosity of the cell colonies were determined through PCR, gel electrophoresis, Sanger sequencing, and droplet digital PCR (ddPCR). Those edited clones that were determined to be homozygous for the desired deletion then underwent macrophage differentiation following the protocol developed by van Wilgenburg et al.^35^. The experimental design is shown in Fig. 3. Along with the CRISPR/Cas9 edited clones, control clones were also taken forward for differentiation. Cell lines henceforth referred to as ‘CRISPR controls’ are cells that underwent the CRISPR/Cas9 transfection process, including gRNA exposure with non-targeting gRNAs, but remained unedited at the target deletion site, and ‘Parental controls’ refer to the unedited iPSC parental cell line that also underwent differentiation (Fig. 3).

### The targeted Intronic region has evidence of enhancer activity for *TNFRSF1A*

**Fig. 4:**
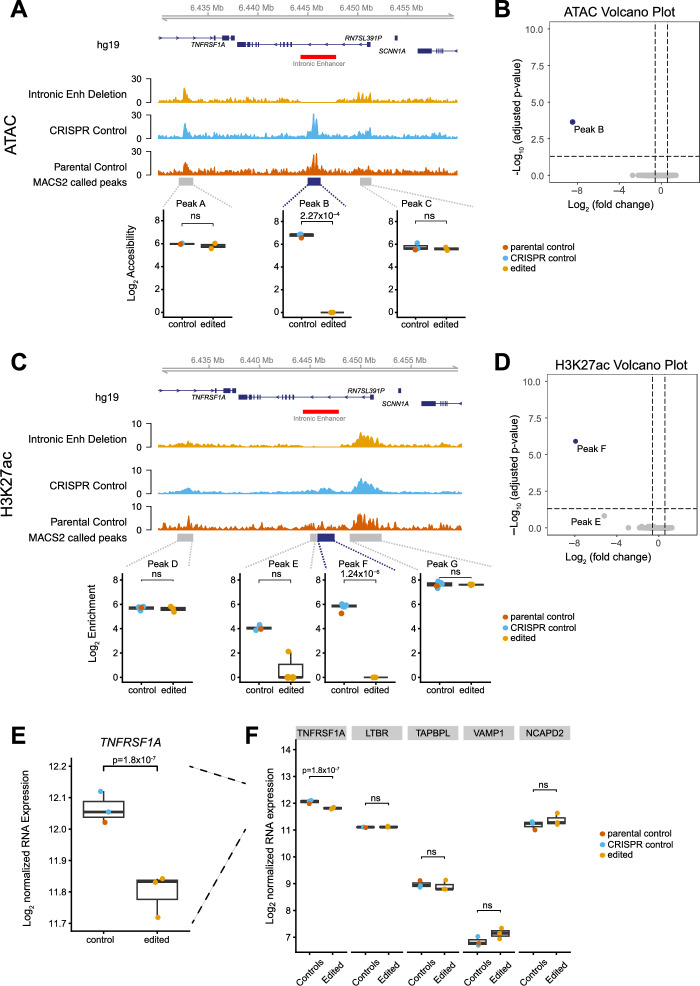
Functional Genomic analysis of CRISPR/Cas9 edited iPSC-derived macrophages (**A**) The differentially open chromatin peak over the Intronic Enhancer Deletion in edited cells and unedited and parental controls. Average tracks of the Intronic Enhancer Deletion clones, CRISPR Controls, and the Parental Control are shown. The significant differential peak B over the Intronic Enhancer is highlighted in dark blue (FDR of 2.27 × 10^−4^). The deletion region is represented by the red box. Boxplots below show DESeq2 Log2 counts for each called peak and significance is DESeq2 padj. (padj > 0.05 ns) (**B**) Volcano plot of all called ATAC peaks showing only Peak B is differential across the whole genome. (**C**) The differential H3K27ac modification peak over the Intronic Enhancer Deletion in edited clones and unedited controls. WashU Epigenome Browser view of the ChIPm read density distribution for edited and unedited iPSC-derived macrophages. Average tracks of Intronic Enhancer Deletion clones, CRISPR Controls, and the Parental Control are shown. The significantly differential peak F over the Intronic Enhancer is highlighted in dark blue with an FDR of 1.24 × 10^−6^. The non-significant peak E has an FDR of 0.15. The deletion region is indicated by the red box. Boxplots below show DESeq2 Log2 counts and significance is DESeq2 padj. (padj > 0.05 ns) (**D**) Volcano plot of all called H3K27ac peaks showing only Peak F is significantly differential across the whole genome. Peak E is not-significant due to a potential read-hop event. (**E**) *TNFRSF1A* expression in edited and unedited iPSC-derived macrophages following a differential analysis between three Intronic Enhancer Deletion edited clones, two CRISPR Controls, and one Parental Control. *TNFRSF1A* was down-regulated in Intronic Enhancer Deletion edited cells compared to the Control cell lines with a Log_2_ fold change of −0.24 and an FDR of 1.8 × 10^–7^. (**F**) Expression of active genes within the TAD containing TNFRSF1A, no other changes are significant.

We then proceeded to use the edited differentiated iPSC macrophage cell lines to investigate whether the epigenetic landscape of the *TNFRSF1A* locus changed as a result of deleting the intronic region. We assayed chromatin accessibility genome-wide in edited cells using ATAC-Seq, as this would also help identify if other off-target ATAC peaks had been affected. As expected, deletion of the intronic element resulted in loss of the peak spanning chr12:6445056–6446217 (edited vs unedited cells FDR of 2.3 × 10^–4^) that colocalizes with the deleted region (Fig. 4A, Table S1). There were no other differential peaks identified in the genome relative to the non-targeted CRISPR clones (Fig. 4B).

We also assayed for effects on histone modifications genome-wide by performing ChIP-Seq for H3K27ac, an active enhancer mark^36,37^. The ChIP-Seq differential analysis between Intronic Enhancer Deletion edited cells and controls showed that the large peak situated at chr12:6445787–6447343 was absent in Intronic Enhancer Deletion edited samples (FDR of 1.24 × 10^–6^) (Fig. 4C, Table S2). This region corresponds to the peak directly over the Intronic Enhancer that was deleted, which is expected. A small second peak called within the Intronic Enhancer is nominally significantly reduced (pvalue of 8.9 × 10^–6^) but is not significant following correction for multiple testing (FDR = 0.15). This is due to a single read pair mapping in one deletion replicate, which is potentially a read-hop event from an unedited sample sequenced on the same flow cell. The input control for the Intronic Enhancer deletion contains no reads within the deleted region. There were no other differential peaks (Fig. 4D), indicating that editing had not resulted in off-target effects within H3K27ac enriched regions.

To better understand the chromatin landscape and the putative enhancers within this genomic region, *TNFRSF1A* was examined for evidence of physical interactions using Capture-C in CD14 + primary cells as Capture-C was not possible in CRISPR/Cas9 edited iPSC-derived macrophages as a minimum of 10 million cells are required for this experiment. The chromatin interactions occurring from the *TNFRSF1A* promoter were interrogated, additionally, one bait for the putative enhancer being examined was also used, Fig. S3. The bait could not be centered on the Intronic Enhancer due to repetitive elements in the DpnII fragment flanks, so the baited fragment is adjacent to it. Peaky^38^ was used to determine which interactions were statistically significant, but it called no interactions in the TAD from the viewpoint of the Intronic Enhancer as being significant using an interaction score of both 0.01 and 0.05. The viewpoint of *TNFRSF1A* promoter showed no interactions with an interaction score less than 0.1.

We then compared gene expression levels between edited cells and CRISPR Controls and the Parental cell line in order to identify any genes regulated by the putative enhancer. The RNA-Seq differential analysis was restricted to the topologically-associating domain (TAD) containing *TNFRSF1A*, as removing an enhancer was anticipated to have the largest effect on genes found within the same TAD. The TAD is 211 kb in size and contains nine protein coding genes, and two non-coding RNA genes^39,40^. It was defined using Hi-C in the B cell-like cell line, GM12878^40^ and TAD boundaries are reported to be relatively invariable across cell types^41^. We found that expression of *TNFRSF1A* was significantly reduced on deletion of the 3.3 kb intronic element with a log_2_ fold change of -0.24 and an FDR of 1.8 × 10^–7^ (Fig. 4E), its expression relative to other actively expressed genes in the TAD is shown in Fig. 4F. The other genes included in this TAD specific analysis were *PLEKHG6*, *SCNN1A*, *LTBR*, *CD27*, *TAPBPL*, *VAMP1*, and *NCAPD2*, and none were differentially expressed (Table S3). qPCR for *TNFRSF1A* did not show a significant difference in expression based on the available replicates (Fig. S4).

## Discussion

It has long been established that TNFα is a key regulator of the inflammatory response and most of its pro-inflammatory functions are mediated through TNFRSF1A^2–4^. However, mechanisms around *TNFRSF1A* gene regulation remain incompletely understood.

We have shown evidence from an iPSC-derived macrophage model for enhancer activity in the first intron of *TNFRSF1A,* through the use of RNA-seq, ChIP-Seq, and ATAC-Seq and luciferase assays. We were unable to show a physical chromatin interaction with the promoter due to the proximity of the enhancer. Consistent with this, a study recently found that the transcription factor ETV7 binds to intron 1 and suppresses *TNFRSF1A* gene expression^25^. They found that both mRNA and protein abundance were reduced in response to ETV7 binding and recruitment of chromatin modulators such as histone methyl-transferases. STAT3 is also known to bind within intron 1 and activate *TNFRSF1A* gene expression^15^. These interactions are considered to compete with one another and modulate the inflammatory response through *TNFRSF1A* transcription and subsequent NF-kB activation^25^. The predicted binding sites for ETV1 and STAT3 fall into the putative enhancer element examined in this work^42^. ETV7 is thought to play a role in breast cancer by down-regulating TNFRSF1A and dampening the immune response^25^. Consistent with our findings, variants within this putative enhancer have been identified as monocyte eQTLs for *TNFRSF1A*^43–45^. Additionally, some of these variants have been associated with immune-mediated disease, including ankylosing spondylitis^7,8^. Understanding the genomic context in which these SNPs lie could bring us closer to deconvoluting complex genetic disease aetiology and uncover effective drug targets.

iPSC-derived macrophages provide a tractable model system to study disease relevant loci and allowed for the initial characterization of a putative regulatory region in a relevant biological system using CRISPR/Cas9. iPSC differentiation between the edited and non-edited controls showed only targeted differences when looking at RNA-seq within the *TNFRSF1A* loci and at the ATAC-seq and ChIP-seq volcano plots (Fig. 4B,D). FACS data was also consistent with differentiation being unaffected by the CRISPR/Cas9 process (Fig. 2B). Due to the mechanism of the latter introducing double stranded breaks, it can lead to cell toxicity and unwanted off target effects^46,47^. The method used here demonstrated clear on-target effects, and therefore the value of this workflow and the generated iPSC lines for functional genomics experiments together with use of epigenetic readouts such as ATAC-Seq and ChIP-Seq to look for off-target effects.

Limitations of the study include the modest effect size observed with the edited cells on gene expression that were significant on RNAseq but not qPCR analysis, likely reflecting limitations in sensitivity of the latter assay. The eQTL findings suggest this may be dependent on stimulation state. Additional replicate edited cell lines would further validate the findings given the relatively small number of replicates used here, together with use of approaches such as CRISPR interference (CRISPRi) and CRISPR activation (CRISPRa)^48–51^. These approaches could also be combined with TNFα exposure or other immune-relevant stimuli in monocyte-derived macrophages to assess if the Intronic Enhancer functions downstream of these pathways and how its role may vary depending on cellular context. This may help clarify the regulatory dynamics of the enhancer as well as its contribution under relevant physiological conditions.

### Experimental procedures

All experiments were performed in accordance with relevant guidelines and regulations.

#### Cell culture and differentiation

The iPSC line used for this project was SFC840-03-03 (AKA AH017-13). It has been previously published^52^ and is deposited in EBiSC (https://cells.ebisc.org/). All experimental protocols were approved by Ethics Committee: National Health Service, Health Research Authority, NRES Committee South Central, Berkshire, UK) (REC 10/H0505/71). The iPSC line was derived from dermal fibroblasts from a male healthy donor recruited through the Oxford Parkinson’s Disease Centre: all subjects were recruited to this study having given signed informed consent, which included derivation of iPSC lines from skin biopsies. iPSCs were cultured in 6-well plates, pre-coated with Matrigel (Catalog# 356234, Corning), in mTeSR media (Catalog# 05850, STEMCELL Technologies) containing 100U/mL penicillin and 0.1 mg/mL streptomycin (Catalog# P0781, Sigma-Aldrich). Rho kinase inhibitor (Catalog# 72302, STEMCELL Technologies) was added during the thawing of the cells to prevent apoptosis. They were grown at 37 °C in 5% CO_2_ and underwent a full media change every day. The cells were passaged every two days using TrypLE (Catalog# 12604013, Gibco). iPSCs were differentiated into macrophages following the protocol laid out by van Wilgenburg et al.^35^.

#### Guide RNA design

CRISPR/Cas9 gRNAs were designed using two online tools. The CRISPR Guide Picker tool designed by Desktop Genetics^53^ was utilized, along with CRISPOR by TEFOR (https://crispor.gi.ucsc.edu). An approximately 100 bp sequence in the desired region of the double stranded break was uploaded onto the software. Both programs selected provided options for gRNAs of around 20 bp in length based on presence of a protospacer adjacent motif (PAM) sequence of NGG, which is required for the Cas9 nuclease to cut the DNA. Both softwares ranked the gRNAs by predicted efficiency using a Doench/Fusi score^54^. These scores are between 0 and 100 and the higher the number, the higher the predicted efficiency. The software also provided information on off target effects by indicating how many other sites in the genome it may bind to, including if one for more nucleotide changes occurred in the genome. The CRISPR/Cas9 gRNAs selected for this project are listed in Table S4.

#### CRISPR/Cas9 genome editing via plasmid method

Complementary oligos of the gRNA sequences (Table 2.2) were ordered and annealed together to generate a small linker with overhangs suitable for cloning into the BbsI site of plasmid pX330-U6-Chimeric BB-CBh-hSpCas9 (Addgene plasmid# 42230) (Cong et al. 2013). These gRNAs were prepared by transforming them into chemically competent *E.coli* cells. DNA was prepared from multiple colonies using the GeneJET Plasmid Miniprep Kit (Catalog# K0502, Thermo Fisher). Colonies were sequenced to verify correct construct insertion and then underwent a midiprep to increase yield using the GeneJET Plasmid Midiprep Kit (Catalog# K0481, Thermo Fisher). gRNAs were tested by transfecting HEK293 cells (Catalog# 85120602, Sigma-Aldrich) and screening for the deletion of interested. Successful gRNAs were carried forward into iPSCs. iPSCs were transfected using a Neon Transfection System (Thermo Fisher) with the 100 µl transfection kit (Thermo Fisher). Prior to transfection, iPSCs were pre-treated with Rho kinase inhibitor (Catalog# 72302, STEMCELL Technologies) and lifted using TrypLE (Catalog# 12604013, Gibco). Cells were counted and 3 × 106 cells were centrifuged at 300 g for 5 min and resuspended in 150µL of Buffer R and mixed with 15 µg of DNA for transfection. Cells were transfected with the following conditions: 1250 V/20 ms/1 pulse. Transfected iPSCs were immediately placed in a 24-well plate containing fresh, pre-warmed mTeSR media (Catalog# 05850, STEMCELL Technologies) and Rho kinase inhibitor (Catalog# 72302, STEMCELL Technologies) that had been pre-coated with Matrigel (Catalog# 356234, Corning). Transfected cells were selected by adding puromycin (Catalog# A1113802, Thermo Fisher) 48 h post transfection at concentrations of 0, 0.1, 0.25, and 0.5 µg/mL for two days.

#### CRISPR/Cas9 genome editing via ribonucleoprotein (RNP) method

gRNAs were ordered as Alt-R CRISPR-Cas9 crRNAs, from Integrated DNA Technologies (IDT) for the transfection of iPSCs. iPSCs were transfected at the Dunn School of Pathology using a Neon Transfection System (Thermo Fisher) with the 10µL transfection kit (Thermo Fisher). Prior to transfection, iPSCs were pre-treated with Rho kinase inhibitor (Catalog# 72302, STEMCELL Technologies) and lifted using TrypLE (Catalog# 12604013, Gibco). The crRNAs and Alt-R® CRISPR-Cas9 tracrRNA (Catalog# 1072532, IDT) were mixed and heated for 5 min at 95 °C, followed by the addition of the Alt-R® S.p. HiFi Cas9 Nuclease (Catalog# 1081060, IDT). The transfection mixture was left to incubate at room temperature for 20 min. iPSCs were counted and 2.2 × 10^5^ cells per transfection were centrifuged at 300 g for 5 min and resuspended in Buffer R. To transfect, RNP transfection solution, 2.2 × 105 iPSCs and Alt-R® Cas9 Electroporation Enhancer (Catalog# 1075915, IDT) were mixed, added to the 10µL tip and transfected with the following conditions: 1250 V/20 ms/1 pulse. Transfected iPSCs were immediately placed in a 24-well plate containing fresh, pre-warmed mTeSR media (Catalog# 05850, STEMCELL Technologies) and Rho kinase inhibitor (Catalog# 72302, STEMCELL Technologies).

#### Clone picking

iPSCs were plated on irradiated mouse embryonic fibroblasts (MEFs) at densities of 500 cells/mL, 1000 cells/mL, and 4500 cells/mL in 2 mL of hES media consisting of KnockOut™ DMEM (Catalog# 10829–018, Life Technologies), KnockOut™ SR (Catalog# 10828–028, Life Technologies), Glutamax (Catalog# 35050, Gibco), NEAA (Catalog# 11140035, Life Technologies), 2-ME (Catalog# 31350–010, Gibco), bFGF (Catalog# 4114-TC-01 M, R&D Systems), with Rho kinase inhibitor (Catalog# 72302, STEMCELL Technologies) in a 6-well plate. Once formed, colonies were picked using a pipette and each colony was transferred to one well of a 96-well plate containing fresh, pre-warmed mTeSR media (Catalog# 05850, STEMCELL Technologies) and Rho kinase inhibitor (Catalog# 72302, STEMCELL Technologies) that had been pre-coated with Matrigel (Catalog# 356234, Corning). Each plasmid transfection was repeated twice, and 96 colonies were selected for screening from each. Approximately 60 clones were selected for screening from each RNP transfection.

#### DNA extraction

DNA was extracted using the Qiagen DNeasy Blood & Tissue Kit (Catalog# 69504) or the Qiagen AllPrep DNA/RNA Kit (Catalog# 80204), following the protocol provided. Briefly, cells were homogenized using the Qiagen QIAshredder (Catalog# 79654) and the nucleic acid was bound to a spin column, where it was washed and reconstituted in Elution buffer. Some large scale, crude DNA extractions were carried out by adding lysis buffer to a plate of colonies and precipitating the DNA using ethanol and NaCl.

#### Luciferase assay

The Intronic Enhancer region was cloned into pGL4.23 luc2/minP vector (Promega, #E841A). K562 cells were seeded in a 24-well plate at 3 × 10^5^ cells per well in 500 µl RPMI-1640 Medium supplemented with 10% FBS (Sigma, F7524) and 1% L-Glut (Thermo, 25030–081) without penicillin–streptomycin. Cells were transfected with 0.1 mg pRL-SV40 (Promega, #E223A) and 1 mg pGL4.23 (Promega, #E841A) with or without the Intronic Enhancer using a ratio of 3:1 FugeneHD (Promega, E2311) to DNA. Cells were assayed for luciferase activity after 48 h using the Dual-Glo® Luciferase Assay System kit (Promega, #E2920) and following the manufacturer’s protocol. Firefly and Renilla luminescence were measured using a CLARIOstar (BMG Labtech). Firefly luciferase activity was normalised against Renilla luciferase activity from three independent experiments with each replica analysed in triplicate.

#### Polymerase chain reaction (PCR)

To determine which clones had incorporated the deletion of interest, PCR was used followed by visualization by gel electrophoresis. The PCR reaction was performed using Platinum Taq DNA Polymerase (Catalog# 10966026, Invitrogen) following the protocol provided by the manufacturer. The desired region was amplified by PCR using primers with binding sites on either side of the desired deletion region to generate PCR products of different sizes, depending on the presence of the deletion. This size difference could then be visualized on a 1% agarose gel. Because smaller PCR products are preferentially amplified, the zygosity of the cells cannot be determined from this PCR because one product is substantially larger. Therefore, another set of primers was designed. Primers sitting just inside the deletion region were designed to amplify the segments of DNA at either side of the deletion. These primers amplify the DNA if a cell population is heterozygous for a deletion but not if it is homozygous. These PCR products are small and similar in size, so amplify with equal efficiency. Additionally, INDELS produced as an artifact of CRISPR/Cas9 editing could encompass the original primer binding site and bias the results, therefore these additional primer sets were used. Primers with binding sites completely internal to the deletion were also designed as an extra check for homozygosity, as they would not amplify in a cell line homozygous for the deletion. The primer sequences used for screening in iPSCs can be seen in Table S5.

#### Sanger sequencing

Sanger sequencing was performed to validate that the correct deletion had occurred after CRISPR/Cas9 editing and to examine how the regions surrounding the deletion joined together. Sequencing was carried out using Mix2Seq kits from Eurofins. The primers used for sequencing were the same primers used for PCR analysis and are listed in Table S5.

#### Droplet digital PCR

Droplet digital PCR (ddPCR) was used to investigate the deletion copy number in expanded cellular clones. Samples were prepared and analyzed following the Copy Number Variation (CNV) Analysis Protocol provided by Bio-Rad. Sample droplets were generated using the QX100 Droplet Generator (Bio-Rad), then amplified using the C1000 Touch Thermal Cycler (Bio-Rad). Visualization of sample droplets was carried out using the QX100 Droplet Reader (BioRad) and analyzed using the QuantaSoft Software (Bio-Rad). RPP30 was used as a copy number reference for all samples. The primers and probes used to examine each deletion are listed in Table S6 and were designed using IDT’s PrimerQuest Tool.

#### Genome integrity check of edited cells

Genome integrity was assessed by Illumina Human OmniExpress24 BeadChip array, evaluating approximately 710,000 markers. DNA from CRISPR/Cas9 edited iPSCs was extracted using the Qiagen DNeasy Blood & Tissue Kit (Catalog# 69504). Analysis was performed using GenomeStudio and Karyostudio software from Illumina using default detection cutoffs. All edited samples were compared to their parental controls and showed no gross abnormalities indicative of affecting their growth or ability to differentiate. This assay detects genomic changes of 1 MB or greater around common variants and an analysis at a higher resolution was not carried out.

#### Flow cytometry

Fluorescence-activated cell sorting (FACS) analysis was performed on each cell type differentiated into macrophages. Approximately 1.5 × 10^6^ cells from each cell line were detached using PBS containing 5 mM EDTA and stained for analysis using antibodies for CD14 and CD11b, or their respective isotype controls. FACS staining was carried out using the following antibodies and controls: PE Mouse anti-human CD14 (HCD14) at 1:20 (Catalog# 325605, Biolegend), PE Mouse IgG1 K Isotype Control (MOPC21) at 1:20 (Catalog# 400113, Biolegend), AlexaFluor-647 Mouse anti-human CD11b (ICRF44) at 1:20 (Catalog# 301319, Biolegend), and AlexaFluor-647 Mouse IgG1 K Isotype Control (MOPC21) at 1:20 (Catalog# 400130, Biolegend).

#### Quantitative PCR

Single strand cDNA was synthesized using Superscript III (Catalog# 18080093, Thermo Fisher), following the protocol provided by the manufacturer. cDNA was synthesized from 1 µg of extracted RNA. qPCR was performed using IQ SybrGreen Supermix (Catalog# 1708880, Bio-Rad), following the protocol provided by the manufacturer. The qPCR was carried out and visualized on the CFX96 Real-Time PCR Detection System (Bio-Rad). *β-actin* was used as the reference gene to normalize C_t_ values and the *β-actin* primer set was developed by Doerner and Zuraw 2009^55^. The *TNFRSF1A* primer set was designed using the Primer3 tool (http://bioinfo.ut.ee/primer3-0.4.0/). Primers used for qPCR analysis are listed in Table S7. Controls prepared without the reverse transcriptase enzyme were also used to check for genomic DNA contamination.

#### RNA-Seq

RNA was extracted using the Qiagen RNeasy Kit (Catalog# 74104) or the Qiagen AllPrep DNA/RNA Kit (Catalog# 80204), following the protocol provided. Sequencing was performed on the Illumina HiSeq4000. RNA-Seq reads were mapped to hg19 using STAR^56^, and sorted and deduplicated with SAMtools^57^ and Picard tools (Broad Institute) to generate unique reads. HTSeq-count^58^ using gencode v29lift37 was then used to generate gene counts. Differential analysis was carried out using DESeq2^59^. Genes with an FDR < 0.05 were considered significant.

#### ATAC-Seq

Omni-ATAC-Seq was carried out following the protocol by Corces et al.^60^. Sequencing was carried out using a high-output kit on the Illumina NextSeq500 to a depth of 25 million paired-end reads. Omni-ATAC-Seq analysis was performed using an in-house pipeline. Sequencing reads were aligned to the hg19 genome build using Bowtie2^61^. Picard tools (Broad Institute) and SAMtools^57^ were used to filter reads. BigWig files were generated to show read pileups for visualization using bedtools^62^. Peak calling and filtering were done using MACS2 callpeak^63^ (parameters: –nomodel, –shift -100, –extsize 200, keep-dup all, call-summits) with a p-value threshold of 1 × 10^–8^ in narrowpeak mode. The median of the summits was used in instances where more than one summit was identified for a peak. Peaks were merged to generate a masterlist and peaks occurring in at least 20% of samples were kept. Differential open chromatin analysis was carried out using DESeq2^59^ in a paired design comparing the Intronic Enhancer deletions against the CRISPR and parental controls. Differential peaks with an FDR < 0.05 were considered significant.

#### ChIP-Seq

ChIP-Seq was carried out following the ChIPmentation (ChIPm) protocol described by Schmidl et al.^64^. Samples were given 2 µg of H3K27ac (Catalog# C15410196, Diagenode) to investigate enhancers. Sequencing was carried out on the Illumina HiSeq4000 to a depth of 25 million paired-end reads. ChIP-Seq analysis was carried out using an in-house ChIPm pipeline. Sequencing reads were aligned to the hg19 genome build using Bowtie2^61^. Picard tools (Broad Institute) and SAMtools^57^ were used to filter reads. BigWig files were generated to show read pileups for visualization using bedtools^62^. Peak calling and filtering was done using MACS2 callpeak^63^ (parameters: –bw200, -p 0.1, keep-dup all, call-summits) with a p-value threshold of 1 × 10^–8^ in narrow peak mode. MACS2 used the average library fragment size (bw) to determine the shift parameter and to find a model to optimally represent locations of precise protein-DNA interactions. Peaks were merged to generate a masterlist and peaks occurring in at least 20% of samples were kept. Differential analysis was carried out using DESeq2^59^. Differential peaks with an FDR < 0.05 were considered significant.

#### Capture-C

Capture-C was carried out on CD14 + primary cells following the protocol described by Davies et al.^65^. Biotinylated baits were designed using CapSequm from the University of Oxford^66^ based on DpnII cut sites. The locations of the genomic regions targeted by the baits can be found in Table S8. Three control regions were added to the baits to test the quality of the Capture-C (CTCF motif in LMO2, CTCF motif near TAL1, and an interaction between RUNX1 and its super enhancer). Sequencing was carried out on the Illumina NextSeq500 using 150 bp paired-end reads. The resulting sequences were analyzed using the ICeCAP pipeline^31^. TrimGalore was used to remove Illumina adapters from the reads and forward and reverse reads were paired using FLASH^67^ and in silico digested at DpnII restriction sites. These sequences were aligned to the hg19 reference genome using Bowtie^61^. The fragments were then classified as either a capture fragment (contains the biotinylated oligo) or a reporter fragment, which represents the ‘other end’ of the interaction. Only interaction read pairs containing a single capture fragment and at least one reporter fragment were kept for analysis. Reporter fragment counts were normalized to the total number of reporters within each sample dataset. Fragments within 1 kb around the capture fragment (exclusion fragments) were removed for clarity. Capture-C interactions were visualized using Gviz^68^. The significance of chromatin interactions was measured using Peaky^38^.

#### Data visualization

Gene expression, chromatin accessibility and histone enrichment were plotted using ggplot2^69^. Genomic views showing RNA-seq, ATAC-Seq, ChIP-Seq and Capture-C data were plotted using Gviz^68^. RNA-seq was plotted into bins of 1kbp using the log2 of the count, directionality is shown with positive and negative counts. ChIP-Seq and ATAC-Seq were plotted with sliding windows of 400 and 200 bp respectively and averaged from all replicates for a given sample.

## Supplementary Information

Supplementary Information 1.

Supplementary Information 2.

Supplementary Information 3.

Supplementary Information 4.

Supplementary Information 5.

Supplementary Information 6.

Supplementary Information 7.

Supplementary Information 8.

Supplementary Information 9.

Supplementary Information 10.

Supplementary Information 11.

## Data Availability

Healthy Control RNA, ATAC, H3K4me3, and H3K27ac data are available from the European Genome-Phenome Archive (EGA) under the accession EGAS00001006233 (https://ega-archive.org/studies/EGAS00001006233). CRISPR and IPSC RNA, ATAC, and H3K27ac/H3K4me3 ChIP data is available under the accession EGAS50000000703 (https://ega-archive.org/studies/EGAS50000000703). BigWigs files for Figs. 1 and 2 are available from Zenodo (10.5281/zenodo.15792065).
