# Supplementary figures and images for: Evidence for enhancer activity in intron 1 of TNFRSF1A using CRISPR/Cas9 in human induced pluripotent stem cell-derived macrophages
Source: Sci Rep. 2025 Oct 7;15:34885. doi: 10.1038/s41598-025-18077-9 (PMC12504462; doi:10.1038/s41598-025-18077-9)

# Supplementary Figure 1

**A**

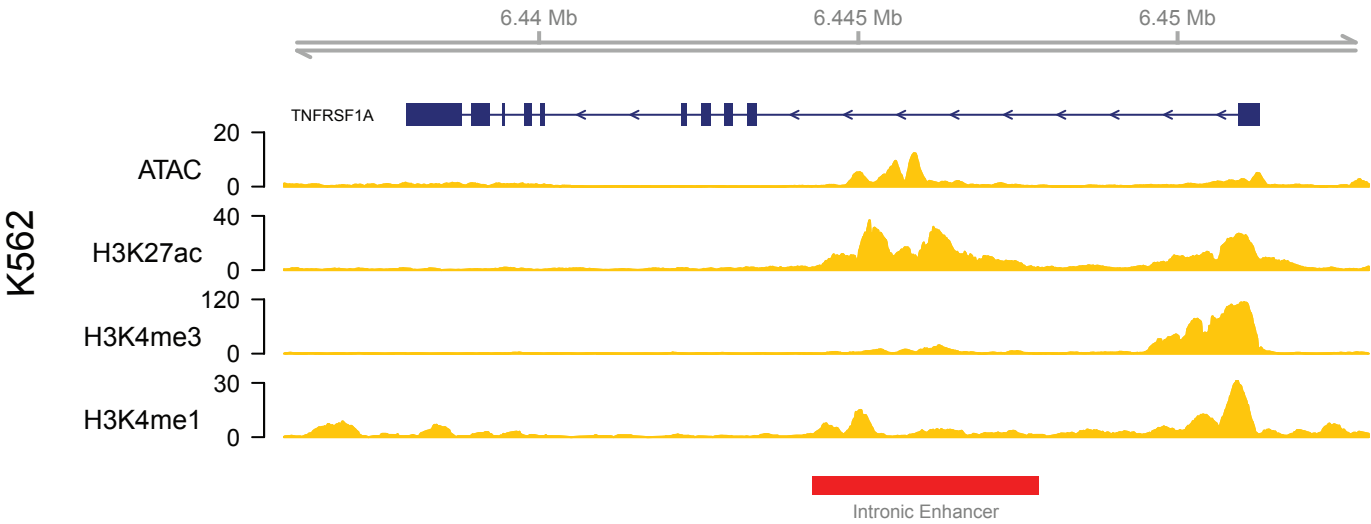

**B**

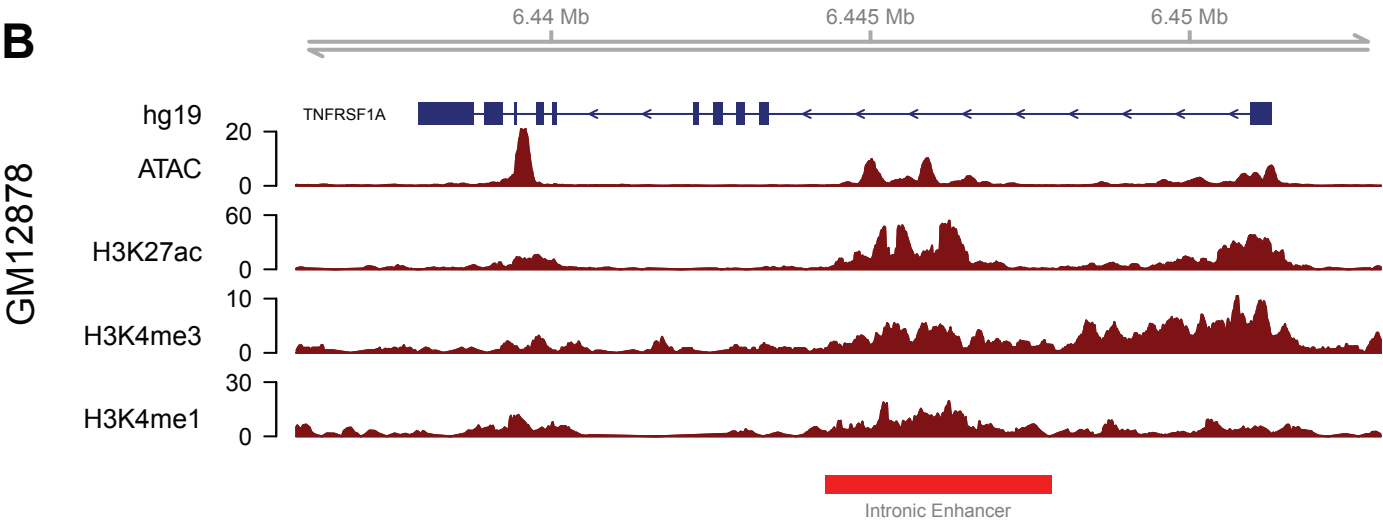

Supplement: Supplementary file 1 — Supplementary Information 1. [file 41598_2025_18077_MOESM1_ESM.pdf]

Supplementary Figure 2

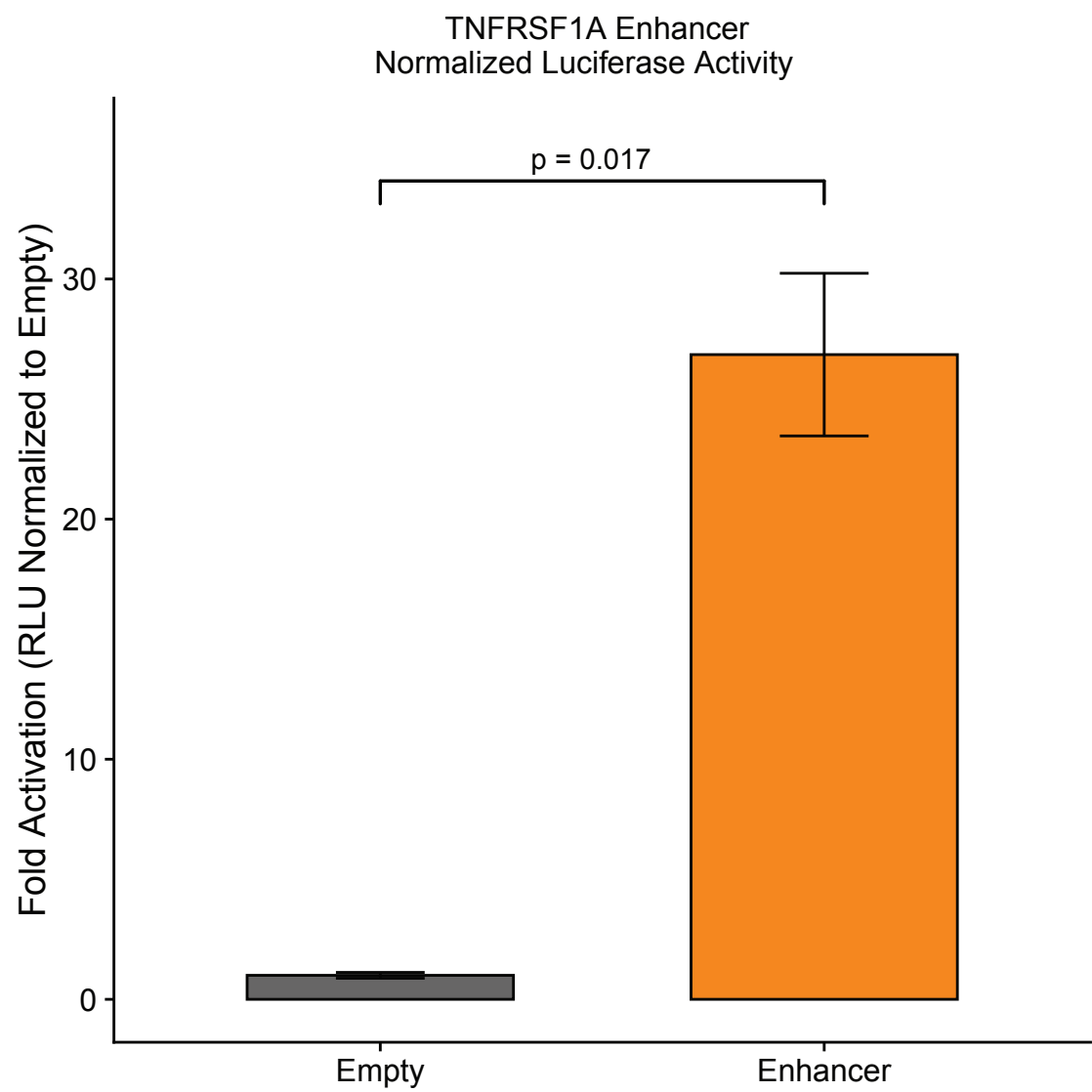

Supplement: Supplementary file 2 — Supplementary Information 2. [file 41598_2025_18077_MOESM2_ESM.pdf]

Supplementary Figure 3

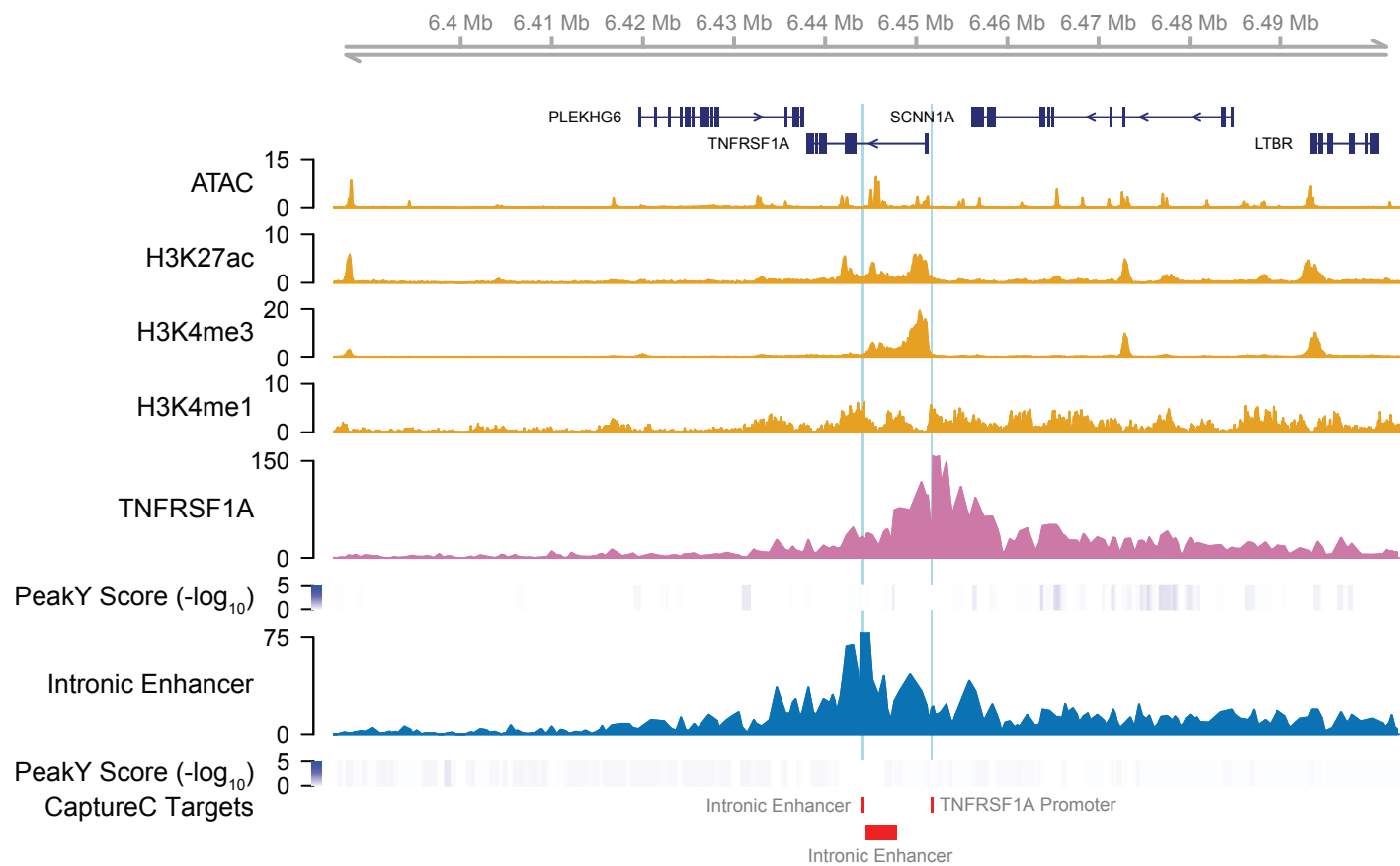

Supplement: Supplementary file 3 — Supplementary Information 3. [file 41598_2025_18077_MOESM3_ESM.pdf]

Supplementary Figure 4

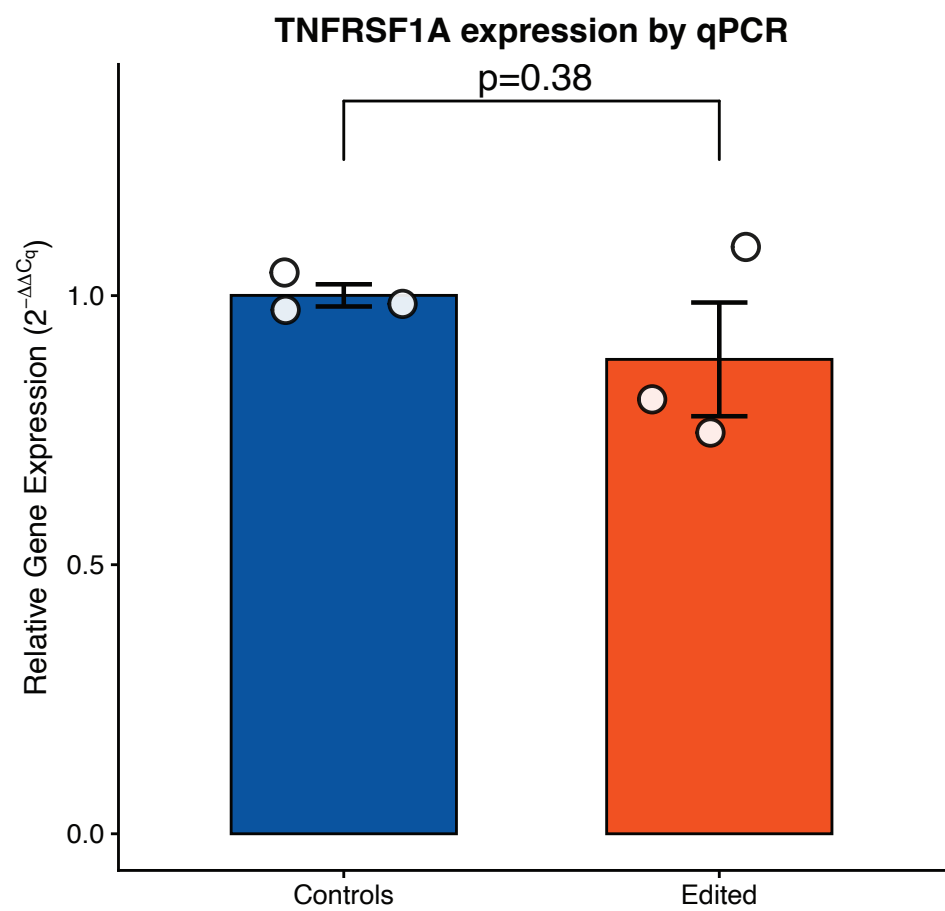

Supplement: Supplementary file 4 — Supplementary Information 4. [file 41598_2025_18077_MOESM4_ESM.pdf]
