# Supplementary material for: Evidence for enhancer activity in intron 1 of TNFRSF1A using CRISPR/Cas9 in human induced pluripotent stem cell-derived macrophages
Source: Sci Rep. 2025 Oct 7;15:34885. doi: 10.1038/s41598-025-18077-9 (PMC12504462; doi:10.1038/s41598-025-18077-9)
Supplement: Supplementary file 5 — Supplementary Information 5. [file 41598_2025_18077_MOESM5_ESM.pdf]

Legends for Supplementary Figures and Tables

Figure S1: Epigenetic landscape of the *TNFRSF1A* locus from Encode. Genomic view of the results of ATAC-Seq, H3K27ac, H3K4me3 and H3K4me1 ChIP in **(A)** K562 myeloid-like leukemia cells and **(B)** GM12878 B-cell-like lymphoblastoid cells. Data for the cell-lines are from Encode.

Figure S2: Enhancer activity of the intronic enhancer. Fold activation of the intronic enhancer assessed by luciferase assay compared to the activity of a minimal promoter. Firefly luciferase activity is normalised to Renilla activity and shown as fold activation over the minimal promoter with background subtracted.

Figure S3: CD14+ monocyte Capture-C data for TNFRSF1A and the intronic enhancer. All the captured fragments were visualized using Gviz and the scales were corrected based on the total number of fragments captured for each sample, visualized with a sliding window of 400bp. Peaks represent interactions between the capture viewpoint and another location. The name listed beside each track denotes the viewpoint. The two tracks labeled PeakY Score show the $-\log_{10}$ interaction score assessed by PeakY for the track above. Capture-C targets show the DpnII fragments used for Capture-C with the blue lines above highlighting these regions across all tracks. The PeakY tracks show no interaction score less than 0.05. Due to the proximity no interaction between TNFRSF1A and the promoter can be assessed.

Figure S4: RT-qPCR analysis of the expression of TNFRSF1A in controls and edited cells. Relative expression of TNFRSF1A calculated by $2^{-\Delta\Delta Cq}$, using B-actin as the control primer. No significant change is seen, p=0.38.

Table S1: Regions of differential open chromatin between Intronic Enhancer Deletion clones and unedited controls. This table lists the peaks called during the Omni-ATAC differential analysis, genome wide. Only one peak was significantly different (FDR of <0.05), which corresponds to the region that was deleted. FDR from DESeq2. The coordinates stated are in UCSC notation based on genome build hg19.

Table S2: Regions of differential chromatin with the H3K27ac histone mark between Intronic Enhancer Deletion clones and unedited controls. This table lists the peaks called during the Omni-ATAC differential analysis, genome wide. Only one peak was significantly different (FDR of <0.05), which corresponds to the region that was deleted. FDR from DESeq2. The coordinates stated are in UCSC notation based on genome build hg19.

Table S3: Differential RNA genes between Intronic Enhancer Deletion clones and unedited controls. This table lists the RNA transcripts, restricted to the *TNFRSF1A* TAD. Only one transcript was significantly different (FDR of <0.05), which corresponds to *TNFRSF1A*. FDR from DESeq2. The coordinates stated are in UCSC notation based on genome build hg19.

Table S4: TNFRSF1A CRISPR/Cas9 gRNAs.

Table S5: Screening primers used to examine TNFRSF1A deletion region in iPSCs. Primers flanking the deletion region were used to amplify the DNA to generate PCR products of a different size to be visualized via gel electrophoresis. Cells with the correct segment of DNA removed would generate a smaller PCR product that those with the region still unedited. To examine the zygosity of the cells, two additional sets of primers were generated. One set of primers sit just within the deleted region and would amplify in a heterozygous cell line but not a homozygous one. They are labeled as ‘Het’ primers. Primers were also designed that bind fully within the deletion region and are labeled as ‘Internal Primers’. They also will not amplify in a cell line homozygous for the deletion of interest.

Table S6: Primers and probes used to examine deletion copy number in CRISPR/Cas9 edited cells by ddPCR. Primers and probes binding within and around the deletion region were designed to examine deletion copy number on cell colonies after CRISPR/Cas9 editing. Cell populations homozygous for the deletion would have no probe binding and no fluorescent signal, resulting in a copy number of zero. The RPP30 gene was used as a reference to calculate copy number in both cell lines. The fluorophores used were FAM for the deletion regions and HEX for RPP30. ZEN and IBFQ were used as quenchers.

Table S7: The qPCR primers used to evaluate *TNFRSF1A* gene expression in iPSC-derived macrophages. *β-actin* was used as reference genes to normalize C_q values.

Table S8: Capture-C bait regions. Genomic coordinates of DpnII fragments targeted by Capture-C bait oligos. The coordinates stated are in UCSC notation based on genome build hg19.
