# Supplementary material for: Evidence for enhancer activity in intron 1 of TNFRSF1A using CRISPR/Cas9 in human induced pluripotent stem cell-derived macrophages
Source: Sci Rep. 2025 Oct 7;15:34885. doi: 10.1038/s41598-025-18077-9 (PMC12504462; doi:10.1038/s41598-025-18077-9)
Supplement: Supplementary file 9 — Supplementary Information 9. [file 41598_2025_18077_MOESM9_ESM.pdf]

Table S4 - CRISPR gRNAs

gRNA Name	Sequence
***TNFRSF1A* Intron Enhancer Deletion 5’**	5’-ACCACCAGGACGGAATGTAG-3’
***TNFRSF1A* Intron Enhancer Deletion 3’**	5’-TCCCCAGGAGTGGTGCTCAC-3’

Table S5 - Screening primers used to examine TNFRSF1A deletion region in iPSCs

Primer name	Sequence
***TNFRSF1A* Intronic Enhancer Deletion**	F 5’-AAAGATTGGGGCGGTGTTTC-3’<br>R 5’-ATCTCAGATGGGTGCTGTGG-3’
***TNFRSF1A* Intronic Enhancer 5’ Het**	F 5’-ACTTCCACAGATGCCTCACT-3’<br>R 5’-ATCTCAGATGGGTGCTGTGG-3’
***TNFRSF1A* Intronic Enhancer 3’ Het**	F 5’-AAAGATTGGGGCGGTGTTTC-3’<br>R 5’-CTAGCCTTGGCCAGCTCTAT-3’
***TNFRSF1A* Intronic Enhancer Deletion Internal**	F 5’-AAGCACGTGAACTGACCCTA-3’<br>R 5’-GAGGAGCACACCAGACTCTT-3’

Table S6 - Primers and probes used to examine deletion copy number in CRISPRCas9 edited cells by ddPCR

Region of Interest	Oligo	Sequence
***TNFRSF1A* Intronic Enhancer Deletion**	F Primer<br>R Primer<br>Probe	5’-GGGTCATGAGTCAGCAGAAA-3’<br>5’-GGCCATAGGAAGAAGGGATTAG-3’<br>5’-CCTGCATGCAAGTGGGACTCCAT-3’
***RPP30* (Reference)**	F Primer<br>R Primer<br>Probe	5’-GATTTGGACCTGCGAGCG-3’<br>5’-GCGGCTGTCTCCACAAGT-3’<br>5’-CTGACCTGAAGGCTCT-3’
